# Editorial Notes

**Published:** 1929

**Authors:** 


### Editorial Notes

**Installation of the Chancellor.**

On Friday, 13th December, the Right Hon. Winston Spencer Churchill, M.P., P.C., C.H., was installed as Chancellor of Bristol University. The installation ceremony took place in the Great Hall, when the Senior Pro-Chancellor, the Right Hon. Henry Hobhouse, P.C., formally announced that Mr. Churchill had been elected Chancellor in succession to the late Lord Haldane, and installed him with due solemnity. The Vice-Chancellor then presented the new Chancellor with a patent of the Degree of Doctor of Laws. Then the Chancellor proceeded to admit to honorary degrees the Right Hon. Miss Margaret Bondfield (Minister of Labour), Admiral Sir Roger Keyes, K.C.B., the Right Hon. Walter Runciman, P.C., M.P., the Right Hon. Philip Snowden (Chancellor of the Exchequer), Dr. Thomas Sibly (Vice-Chancellor of the University of Reading), Mr. Walter de la Mare, and Dr. Vaughan Williams. In the evening the members of Council and Senate, together with the honorary graduates, dined in the Reception Room at the University, under the Chairmanship of the Chancellor, Mrs. Churchill being present also.

On the morning of Saturday, 14th December, Mr. Churchill addressed the undergraduates at the Union Club (Victoria Rooms), and then proceeded to Wills Hall, which he declared formally "open." In the evening a reception was held in the University and

Art Gallery, at which guests to the number of 2,000 or more were present.

The new Chancellor was in his best form. He gave the impression that he was thoroughly enjoying himself, and whilst he could be dignified enough when occasion required, he diffused an atmosphere of geniality and wit which gave promise that in his capacity of University Chancellor his personality would not be subdued by the weight of his academic robes and the golden tassel of his college cap.

**Colston Research Society.**

BY a splendid gift of £5,000 Mr. R. H. Mardon has created a new position for the Colston Research Society. It will be necessary for the Society to obtain a charter of incorporation so that it may have a proper legal status and be empowered to hold trust funds. Mr. Mardon has added a further £100 to his gift to meet the cost of incorporation. This is an encouraging recognition of a Society which has done a great deal for the University, and it is to be hoped that its activities will now be more widely appreciated by the public.

The University College Colston Society, as it was then called, came into existence as the result of an article by the late Mr. Walter Reid, in the *Western Daily Press*, urging the formation of a Society conceived on the lines of the existing Colston Societies—the Dolphin, the Grateful and the Anchor—but to commemorate the educational work of Edward Colston rather than the philanthropic by supporting the cause of higher education in Bristol and Bristol University College in particular. It was founded in 1899 largely through the initiative and energy of the late Mr. J. W. Arrowsmith, who acted as Hon. Secretary and

Treasurer to the day of his death. The Society played a big part in arousing public interest in University College. The President for the year made a collection to assist the funds of the University College, and presided at the Annual Dinner, at which some distinguished guest spoke in the cause of University education.

When in the course of a few years the idea of a University for Bristol began to emerge into the region of practical politics, Mr. Arrowsmith directed all the influence and activity of the Colston Society towards this goal. In 1909 the University obtained its Charter, and the one aim of the Society was achieved. Mr. Arrowsmith was disinclined to let so useful an organization dissolve because success had followed its efforts in one particular direction. A new objective was set, namely to appeal for funds for the support and furtherance of research work in the University. When James Arrowsmith died in 1913 the secretarial work of the Society was taken up by his nephew, James Arrowsmith-Brown, under whom there has been no relaxing of energy. Public interest has been still further stimulated, and the commercial and industrial community have given increasing support to the appeal of the Society for funds to enable research work to be undertaken. Altogether the sum of over £15,000 has been raised by the Society, apart from Mr. Mardon's gift, and applied first in the cause of higher education and then of research. Hitherto the Society has only had its annual contributions to allocate and distribute; now Mr. R. H. Mardon's gift has put the Society in the embarrassing position of owning trust funds, and in order that it may legally (and all Statutes of Mortmain notwithstanding) hold and administer such funds the Society must become Incorporated.

